# Post-Marketing Safety Concerns With Secukinumab: A Disproportionality Analysis of the FDA Adverse Event Reporting System

**DOI:** 10.3389/fphar.2022.862508

**Published:** 2022-06-08

**Authors:** Yamin Shu, Yufeng Ding, Yanxin Liu, Pan Wu, Xucheng He, Qilin Zhang

**Affiliations:** ^1^ Department of Pharmacy, Tongji Hospital, Tongji Medical College, Huazhong University of Science and Technology, Wuhan, China; ^2^ Department of Pharmacy, Pengzhou People’s Hospital, Pengzhou, China; ^3^ Department of Pharmacy, Qionglai Maternal and Child Health and Family Planning Service Center, Qionglai, China; ^4^ Department of Pharmacy, Pengzhou Second People’s Hospital, Pengzhou, China; ^5^ Department of Pharmacy, Union Hospital, Tongji Medical College, Huazhong University of Science and Technology, Wuhan, China

**Keywords:** adverse event, data mining, FAERS, pharmacovigilance, secukinumab

## Abstract

**Purpose:** Secukinumab was approved for the treatment of psoriasis, psoriatic arthritis, and ankylosing spondylitis. However, the long-term safety of secukinumab in large sample population was unknown. The current study was to evaluate the secukinumab-assocaited adverse events (AEs) through data mining of the US Food and Drug Administration Adverse Event Reporting System (FAERS).

**Methods:** Reports in the FAERS from the first quarter of 2015 (FDA approval of secukinumab) to the third quarter of 2021 were collected and analyzed. Disproportionality analyses, including the reporting odds ratio (ROR), the proportional reporting ratio (PRR), the Bayesian confidence propagation neural network (BCPNN), and the multi-item gamma Poisson shrinker (MGPS) algorithms, were employed in data mining to quantify the signals of secukinumab-related AEs.

**Results:** A total of 89,228 reports of secukinumab as the “primary suspected (PS)” and 254,886 AEs induced by secukinumab were identified. Secukinumab-induced AE occurrence targeted 27 system organ classes (SOCs). A total of 257 signals of secukinumab-induced AEs in 19 SOCs were detected after conforming to the four algorithms simultaneously. Common significant signals of infections, respiratory disorders, skin and subcutaneous tissue disorders, immune system disorders, and ear and labyrinth disorders have emerged. Unexpected significant AEs such as injection site pain, vessel puncture site haemorrhage, arthralgia, hypokinesia, Bell’s palsy, parotid gland enlargement, and stress might also occur. The median onset time of secukinumab-associated AEs was 56 days (interquartile range [IQR] 5–214 days), and most of the onsets occurred within the first 1, 2, 3, and 4 months after initiation of secukinumab.

**Conclusion:** Our study found potential new AE signals and provided a broader understanding of secukinumab’s safety profiles, supporting its rational use in chronic systemic inflammatory diseases.

## Introduction

Psoriasis (PsO) is an immune-mediated chronic, recurrent, inflammatory skin disease (Frieder et al., 2018). Analysis of epidemiological data from 40 countries showed that the prevalence of PsO in adults was about 0.51–11.43% and in children was 0–1.37% (Michalek et al., 2017). There are approximately 125 million PsO patients worldwide, with plaque psoriasis accounting for 90% (Armstrong and Read, 2020). The pathogenesis of PsO may involve heredity, immunity and environment, and PsO may be caused by the immune response mediated by T lymphocytes and other various immune cells, leading to excessive proliferation of keratinocytes and inflammation of synovial cells and chondrocytes in joints (Krueger et al., 2012).

Currently, there is no cure for PsO. Tumor necrosis factor α (TNF-α) antagonists have been the traditional biological treatment for moderate-to-severe plaque psoriasis (Campa et al., 2015). However, with great progress in the understanding of this disease, new drugs targeting the interleukin (IL), such as the IL-23/IL-17 pathway, have emerged, significantly improving the quality of life of PsO patients (Di Cesare et al., 2009; Rønholt and Iversen, 2017). Studies have shown that the new generation of monoclonal antibodies, such as IL-12/23, IL-23, and IL-17A inhibitors, could reverse the clinical, histological, and molecular characteristics of PsO in approximately 70–80% of patients, compared with only 45–50% with TNF-α antagonists (Chiricozzi and Krueger, 2013; Levin and Gottlieb, 2014). IL-17A has been identified as a key driver of pro-inflammatory cytokines in PsO pathogenesis (Helliwell and Coates, 2015; Mease et al., 2015; Ryoo et al., 2016).

Secukinumab is a recombinant, high-affinity fully humanized immunoglobulin G1 (IgG1)/κ monoclonal antibody that selectively targets IL-17A and blocks its interaction with the IL-17A receptor, thereby inhibiting the release of pro-inflammatory cytokines (i.e., TNF-α, IL-6 and IL-1β) and chemokines (i.e., CCL20, CXCL1 and CXCL8), interfering the key pathway of PsO while promoting normalization of immune function and skin histology (Harper et al., 2009; Frieder et al., 2018; Mease et al., 2018). Neutralization of IL-17A by secukinumab opened up a new strategy for the treatment of plaque psoriasis. Secukinumab was approved by the US Food and Drug Administration (FDA) in January 2015 for the treatment of moderate-to-severe plaque psoriasis (Fala, 2016). Subsequently, it was approved for the treatment of psoriatic arthritis (PsA), ankylosing spondylitis (AS), and non-radioactive axial spinal arthritis due to its distinct anti-inflammatory and anti-rheumatism efficacy (Frieder et al., 2018; Blair, 2019).

Despite its late introduction, secukinumab has been widely used in clinic due to its rapid onset of action, improved efficacy, and sustained long-term clinical responses (Ryoo et al., 2016). Reports of related adverse events (AEs) have gradually increased. Moreover, off-label adverse drug reactions (ADRs) have also appeared, such as injection site reactions reported by Grace et al. (2020). However, the safety data of secukinumab are mostly reported in short-term clinical trials, case reports, or meta-analyses, and ADRs are often concentrated on a single or multiple systems due to the strict diagnostic and selection criteria (Augustin et al., 2020; Zhou et al., 2020). The sample size is relatively small, and the follow-up duration and observable AEs are limited. In addition, long-term use of secukinumab may present previously unrecognized or serious safety concerns (Jin et al., 2021). Moreover, the time to onset of AEs was usually unknown. Therefore, it is of great significance and necessity to explore the potential ADR signals of secukinumab through data mining algorithm by large-sample post-marketing monitoring.

The FDA Adverse Event Reporting System (FAERS) database is a publicly accessible spontaneous reporting system (SRS), which covers tens of millions of case reports of adverse drug events (ADEs) submitted by physicians, pharmacists, manufacturers, and others (Grace et al., 2020). The FAERS is currently the world’s largest pharmacovigilance database and is an effective tool for detecting ADRs associated with drug exposure. In the present study, we aimed to evaluate AEs of secukinumab by post-marketing through FAERS data mining to provide a reference for its clinical monitoring and risk identification.

## Methods

### Data Source

**FIGURE 1:**
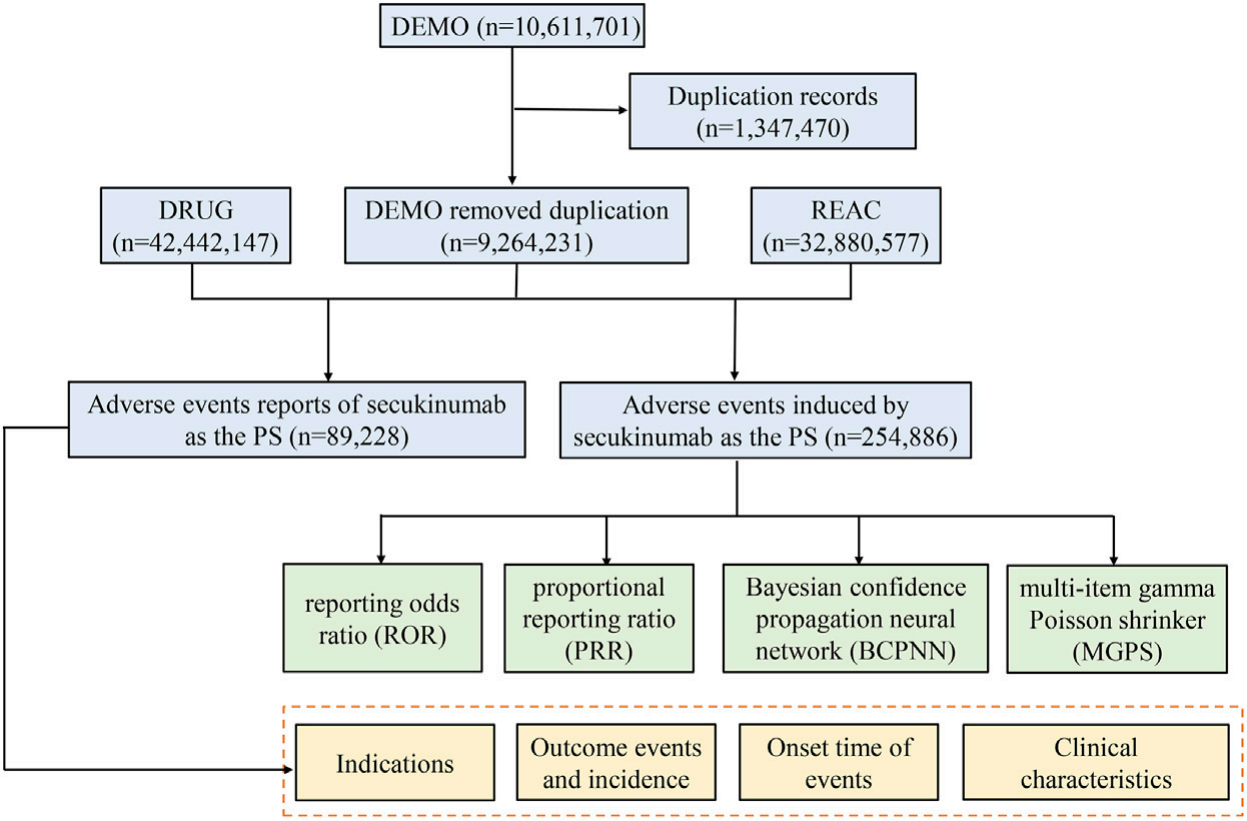
The process of selecting secukinumab-associated adverse events from Food and Drug Administration adverse event reporting database.

We performed a retrospective pharmacovigilance study using data from the FAERS database covering the period from the first quarter of 2015 (FDA approval of secukinumab) to the third quarter of 2021 (the most recent update of the FAERS database at the time the study was conducted). The FAERS data files contained seven types of datasets: patient demographic and administrative information (DEMO), drug information (DRUG), coded for the adverse events (REAC), patient outcomes (OUTC), report sources (RPSR), therapy start dates and end dates for reported drugs (THER), and indications for drug administration (INDI), and deleted cases. All the data downloaded from the U.S. FDA website were imported into MySQL 8.0 for further analysis. A total of 10,611,701 reports were obtained from the FAERS database. Since the database is updated quarterly, it will inevitably duplicate the previous public reports, so it needs to be reprocessed. The deduplication process is performed before statistical analysis according to FDA’s recommendations, by selecting the latest FDA_DT when CASEID were the same, and choosing the higher PRIMARYID when the CASEID and FDA_DT were the same, resulting in a reduction in the number of reports to 9,264,231 (Figure 1).

### Adverse Event and Drug Identification

AEs in the FAERS database were coded by Medical Dictionary for Regulatory Activities 24.0 (MedDRA). The structural hierarchy of the MedDRA terminology was divided into five levels: system organ class (SOC), high-level group term (HLGT), high-level term (HLT), preferred term (PT), and lowest-level term (LLT) (Mascolo et al., 2021). All AEs of secukinumab reports taken from the REAC files in the FAERS database were identified to describe the frequency and intensity based on MedDRA at SOC and PT levels in our study. FAERS permits the reporting of arbitrary drug names approved by the FDA, and drug names in our study were classified into generic name and brand name including secukinumab and cosentyx, respectively, by using IBM Micromedex as the dictionary. The role_code about AEs had been assigned by reporters, including primary suspected (PS), secondary suspect drug (SS), concomitant (C), and interacting (I). In order to improve accuracy, we choose the role_cod as“PS” (primary suspected) in the DRUG files.

### Data Mining

The disproportionality analysis is regarded as a fundamental tool of analytic methods in pharmacovigilance study to identify drug-associated AEs as signals, which compares the proportion of occurring AEs between a specific drug and all other drugs (Hu et al., 2020). The general principle is that a signal is considered to have been generated in the data extraction period, when the specific AE occurrence rate of a specific drug is significantly higher than the background frequency in the database and reaches a certain threshold or criteria. Both Frequentist and Bayesian methods in the disproportionality analysis were employed to investigate the association between a drug and an AE, by using the reporting odds ratio (ROR), the proportional reporting ratio (PRR), the information component (IC) and the empirical bayes geometric mean (EBGM) (van Puijenbroek et al., 2002; Song et al., 2020). In this study, the AEs signals could be detected when conforming to the four algorithm criteria simultaneously. The equations and criteria for the four algorithms are shown in Supplementary Table S1.

The time to onset and serious outcome probability of AEs were calculated. The onset time is defined as the interval between EVENT_DT (date of AE occurrence) and START_DT (start date for secukinumab use). In addition, reports with input errors (EVENT_DT earlier than START_DT), inaccurate date entries and missing specific data were excluded. Severe outcomes mainly included life-threatening events or those causing hospitalization, disability, or death. Moreover, reports with serious outcome events attributed to drug toxicity were counted, and the proportion was calculated as dividing the number of serious outcomes by the total number of reported events. All data processing and statistical analyses were performed using MYSQL 8.0, Navicat Premium 15, Microsoft EXCEL 2019 and the GraphPad Prism 8 (GraphPad Software, CA, United States).

## Results

### Descriptive Analysis

From January 2015 to September 2021, a total of 9,264,231 reports were documented in the FAERS database after the exclusion of duplicates. There were 89,228 reports of secukinumab as the PS and 254,886 AEs induced by secukinumab as the suspected drug were identified. The clinical characteristics of events with secukinumab were described in Table 1. The number of AE reports with secukinumab had gradually increased from 2015 to 2021. Among all reports, females (56.32%) accounted for a larger proportion than males (38.21%). Patients were mainly aged >18 years old in the reports recording age, with a median age of 54. Most of reports were came from America (34.64%), followed by Canada (2.28%), Britain (1.46%), Germany (1.42%), and Japan (0.71%), mainly submitted by consumers (62.55%). Psoriasis (40.20%), psoriatic arthropathy (19.04%) and ankylosing spondylitis (7.26%) were the most reported indications, which were consistent with indications approved by FDA. Hospitalization (8.12%) was the most frequently reported serious outcome, and death or life-threatening events were reported in 1438 (1.61%) and 693 (0.78%) cases, respectively.

**TABLE 1:** Clinical characteristics of reports with secukinumab from the FAERS database (January 2015 to September 2021).

Characteristics	Case number, *n*	Case proportion, %
Number of events	89,228	
Gender		
Female	50,251	56.32
Male	34,098	38.21
Unknown	4,879	5.47
Age (years)		
<18	113	0.13
18≤ and ≤65	27,556	30.88
>65	5,539	6.21
Unknown	56,020	62.78
Indications (TOP five)		
Psoriasis	35,870	40.20
Psoriatic arthropathy	16,991	19.04
Ankylosing spondylitis	6,474	7.26
Rheumatoid arthritis	401	0.45
Pustular psoriasis	158	0.18
Serious Outcome		
Death	1,438	1.61
Life-threatening	693	0.78
Hospitalization	7,247	8.12
Disability	560	0.63
Congenital anomaly	22	0.02
Required intervention to prevent permanent impairment/damage	18	0.02
Other serious medical events	18,528	20.76
Reported Countries (Top five)		
America	30,913	34.64
Canada	2,034	2.28
Britain	1,303	1.46
Germany	1,270	1.42
Japan	632	0.71
Reported Person		
Health profession		
Physician	12,918	14.48
Pharmacist	3,517	3.94
Other health professional	14,665	16.43
Non-healthcare professional		
Consumer	55,809	62.55
Unknown	2,319	2.60
Reporting year		
2021 Q3^a^	15,358	17.21
2020	19,958	22.37
2019	17,834	19.99
2018	18,142	20.33
2017	10,097	11.32
2016	5,409	6.06
2015	2,430	2.72

a The third quarter of 2021.

### Disproportionality Analysis

Signal strengths and reports of secukinumab at the System Organ Class (SOC) level were described in Table 2. Statistically, we found that secukinumab-induced AEs occurrence targeted 27 SOCs. The significant SOCs that at least one of the four algorithm meets the criteria were general disorders and administration site conditions (SOC: 10018065, 40825), skin and subcutaneous tissue disorders (SOC: 10040785, 30047), immune system disorders (SOC: 10021428, 20965), infections and infestations (SOC: 10021881, 19387), musculoskeletal and connective tissue disorders (SOC: 10028395, 17294), respiratory, thoracic and mediastinal disorders (SOC: 10038738, 15948), ear and labyrinth disorders (SOC: 10013993, 1610).

**TABLE 2:** Signal strength of reports of secukinumab at the System Organ Class (SOC) level in the FAERS database.

System organ class (SOC)	Secukinumab cases reporting SOC	ROR (95%two-sided CI)	PRR (χ2)	IC (IC025)	EBGM (EBGM05)
General disorders and administration site conditions	40,825	1.30 (1.29–1.32)^a^	1.17 (1559.78)	0.22 (0.20)^a^	1.16 (1.15)
Skin and subcutaneous tissue disorders	30,047	2.65 (2.61–2.69)^a^	2.09 (20096.13)^a^	1.05 (1.03)^a^	2.07 (2.04)^a^
Injury, poisoning and procedural complications	24,402	0.93 (0.91–0.94)	0.95 (101.12)	−0.08 (−0.10)	0.95 (0.93)
Immune system disorders	20,965	3.03 (2.98–3.07)^a^	2.55 (21257.54)^a^	1.33 (1.31)^a^	2.51 (2.47)^a^
Infections and infestations	19,387	2.25 (2.21–2.29)^a^	1.98 (10340.17)	0.97 (0.95)^a^	1.96 (1.93)
Musculoskeletal and connective tissue disorders	17,294	1.69 (1.66–1.72)^a^	1.56 (3882.64)	0.63 (0.61)^a^	1.55 (1.52)
Respiratory, thoracic and mediastinal disorders	15,948	1.29 (1.27–1.32)^a^	1.24 (859.01)	0.31 (0.28)^a^	1.24 (1.22)
Gastrointestinal disorders	12,951	0.76 (0.74–0.77)	0.79 (842.05)	−0.33 (−0.36)	0.80 (0.78)
Nervous system disorders	11,740	0.55 (0.54–0.56)	0.61 (3645.60)	−0.70 (−0.73)	0.61 (0.60)
Vascular disorders	8,642	0.56 (0.55–0.58)	0.61 (2604.10)	−0.72 (−0.75)	0.61 (0.60)
Psychiatric disorders	6,381	0.52 (0.50–0.53)	0.55 (2669.14)	−0.85 (−0.89)	0.55 (0.54)
Cardiac disorders	6,175	0.55 (0.54–0.57)	0.58 (2071.19)	−0.77 (−0.81)	0.59 (0.57)
Investigations	5,453	0.49 (0.48–0.50)	0.52 (2711.49)	−0.93 (−0.98)	0.52 (0.51)
Product issues	3,670	0.98 (0.95–1.01)	0.98 (1.46)	−0.03 (−0.08)	0.98 (0.95)
Renal and urinary disorders	3,261	0.53 (0.51–0.55)	0.55 (1298.92)	−0.86 (−0.91)	0.55 (0.53)
Metabolism and nutrition disorders	2,734	0.40 (0.39–0.42)	0.42 (2320.40)	−1.24 (−1.29)	0.42 (0.41)
Neoplasms benign, malignant and unspecified (incl cysts and polyps)	2,709	0.41 (0.39–0.42)	0.42 (2280.68)	−1.23 (−1.29)	0.43 (0.41)
Eye disorders	2,492	0.64 (0.62–0.67)	0.65 (474.18)	−0.61 (−0.67)	0.66 (0.63)
Reproductive system and breast disorders	1,715	0.45 (0.43–0.47)	0.46 (1127.11)	−1.11 (−1.18)	0.46 (0.44)
Ear and labyrinth disorders	1,610	1.40 (1.33–1.47)^a^	1.39 (179.15)	0.47 (0.40)^a^	1.39 (1.32)
Blood and lymphatic system disorders	1,293	0.29 (0.27–0.31)	0.30 (2219.18)	−1.73 (−1.81)	0.30 (0.29)
Surgical and medical procedures	1,288	0.42 (0.40–0.44)	0.43 (1008.86)	−1.21 (−1.29)	0.43 (0.41)
Hepatobiliary disorders	1,130	0.48 (0.46–0.51)	0.49 (609.16)	−1.02 (−1.11)	0.49 (0.47)
Endocrine disorders	1,117	0.47 (0.44–0.50)	0.48 (648.03)	−1.06 (−1.14)	0.48 (0.45)
Pregnancy, puerperium and perinatal conditions	671	0.41 (0.38–0.45)	0.42 (549.87)	−1.25 (−1.36)	0.42 (0.39)
Social circumstances	220	0.21 (0.19–0.24)	0.22 (632.00)	−2.20 (−2.40)	0.22 (0.19)
Congenital, familial and genetic disorders	95	0.18 (0.15–0.22)	0.18 (346.84)	−2.44 (−2.73)	0.18 (0.15)

a Indicates statistically significant signals in algorithm.

ROR, reporting odds ratio; CI, confidence interval; PRR, proportional reporting ratio; χ^2^, chi-squared; IC, information component; IC025, the lower limit of 95% CI of the IC; EBGM, empirical Bayesian geometric mean; EBGM05, the lower limit of 95% CI of EBGM.

A total of 257 signals of secukinumab-induced AEs in 19 SOCs were detected after conforming to the four algorithms simultaneously. The number of reporting PTs >100 were showed in Table 3, including 87 PTs and 11 corresponding SOCs, and others were listed in Supplementary Table S2. In our study, PTs of nasopharyngitis (PT: 10028810), upper respiratory tract infection (PT: 10046306), pharyngitis (PT: 10034835), lower respiratory tract infection (PT: 10024968), rhinorrhoea (PT: 10039101), oral herpes (PT: 10067152), oral candidiasis (PT: 10030963), inflammatory bowel disease (PT: 10021972), external ear inflammation (PT: 10065837), middle ear inflammation (PT: 10065838), skin exfoliation (PT: 10040844), C-reactive protein increased (PT: 10006825) and uveitis (PT: 10046851) were presented, which were indicated in the label for secukinumab. Of note, a lot of unexpected significant AEs that uncovered in the label were found in our data mining, such as PTs of vessel puncture site haemorrhage (PT: 10054092), injection site nerve damage (PT: 10022083), hypokinesia (PT: 10021021), anosmia (PT: 10002653), genital ulceration (PT: 10018180), musculoskeletal stiffness (PT: 10052904), joint swelling (PT: 10023232) and arthralgia (PT: 10003239). Hypercholesterolaemia (PT: 10020603), neutrophil count decreased (PT: 10029366) and transaminases increased (PT: 10054889), which were listed in the drug label, didn’t meet the criteria for at least one of the four algorithms.

**TABLE 3:** Signal strength of reports of secukinumab at the Preferred Term (PT) level in the FAERS database.

SOC	Preferred terms (PTs)	Secukinumab cases reporting PT	ROR (95%two-sided CI)	PRR (χ2)	IC (IC025)	EBGM (EBGM05)
Eye disorders	Uveitis	281	5.90 (5 23–6.65)	5.88 (1077.70)	2.49 (2.28)	5.62 (4.98)
Gastrointestinal disorders	Irritable bowel syndrome	245	3.56 (3.14–4.05)	3.55 (435.15)	1.79 (1.59)	3.47 (3.05)
	Inflammatory bowel disease	224	11.10 (9.67–12.75)	11.08 (1854.59)	3.34 (3.07)	10.1 (8.80)
	Mouth ulceration	189	2.35 (2.03–2.71)	2.34 (142.66)	1.21 (0.98)	2.31 (2.00)
	Aphthous ulcer	131	3.54 (2.98–4.22)	3.54 (230.92)	1.79 (1.49)	3.46 (2.90)
	Drug ineffective	13,472	2.40 (2.35–2.44)	2.19 (9132.22)	1.11 (1.09)	2.16 (2.12)
	Pain	6,372	2.73 (2.66–2.80)	2.61 (6326.11)	1.36 (1.32)	2.57 (2.50)
	Therapeutic product effect incomplete	2,314	4.00 (3.84–4.17)	3.92 (4886.46)	1.93 (1.87)	3.82 (3.66)
	Injection site bruising^a^	1,204	3.47 (3.28–3.68)	3.44 (2022.96)	1.75 (1.66)	3.36 (3.17)
	Inflammation	922	4.95 (4.63–5.29)	4.91 (2745.91)	2.24 (2.14)	4.73 (4.43)
	Illness	600	3.39 (3.13–3.68)	3.38 (974.85)	1.72 (1.60)	3.30 (3.04)
	Therapeutic response shortened	595	7.60 (6.99–8.27)	7.56 (3157.82)	2.83 (2.69)	7.11 (6.54)
	Concomitant disease aggravated	320	9.84 (8.77–11.04)	9.81 (2312.37)	3.18 (2.97)	9.04 (8.06)
	Therapeutic product effect delayed	155	3.50 (2.98–4.11)	3.50 (267.47)	1.77 (1.50)	3.42 (2.91)
	Tenderness	146	3.41 (2.89–4.02)	3.40 (239.69)	1.73 (1.46)	3.32 (2.82)
	Secretion discharge^a^	144	2.62 (2.22–3.09)	2.62 (140.45)	1.37 (1.10)	2.58 (2.18)
	Symptom recurrence	106	10.18 (8.34–12.43)	10.17 (797.44)	3.22 (2.81)	9.34 (7.65)
Immune system disorders	Decreased immune responsiveness	497	12.62 (11.49–13.85)	12.55 (4711.97)	3.50 (3.33)	11.30 (10.29)
	Immune system disorder	201	3.50 (3.04–4.03)	3.50 (346.84)	1.77 (1.54)	3.42 (2.97)
Infections and infestations	Nasopharyngitis	3,014	3.97 (3.83–4.12)	3.87 (6235.37)	1.91 (1.86)	3.76 (3.63)
	Influenza	1,606	3.31 (3.15–3.48)	3.27 (2467.92)	1.68 (1.60)	3.20 (3.04)
	Sinusitis	1,541	3.72 (3.53–3.92)	3.67 (2908.82)	1.84 (1.76)	3.58 (3.40)
	Infection	1,334	2.30 (2.18–2.43)	2.28 (944.42)	1.17 (1.09)	2.25 (2.13)
	COVID-19^a^	1,267	4.11 (3.88–4.35)	4.06 (2823.94)	1.98 (1.89)	3.95 (3.73)
	Lower respiratory tract infection	942	4.83 (4.53–5.16)	4.79 (2707.23)	2.21 (2.11)	4.62 (4.33)
	Bronchitis	774	2.34 (2.17–2.51)	2.32 (573.13)	1.20 (1.09)	2.29 (2.14)
	Upper respiratory tract infection	747	3.85 (3.58–4.14)	3.82 (1505.86)	1.90 (1.78)	3.72 (3.46)
	Cellulitis	672	3.16 (2.93–3.41)	3.14 (955.38)	1.62 (1.50)	3.08 (2.85)
	Ear infection	481	4.66 (4.25–5.11)	4.64 (1316.04)	2.16 (2.02)	4.48 (4.09)
	Fungal infection	408	2.81 (2.54–3.10)	2.80 (459.62)	1.46 (1.30)	2.75 (2.49)
	Candida infection	364	4.57 (4.11–5.07)	4.55 (966.93)	2.14 (1.97)	4.40 (3.96)
	Staphylococcal infection	361	2.93 (2.63–3.25)	2.92 (443.26)	1.52 (1.35)	2.87 (2.58)
	Localised infection	319	2.97 (2.65–3.32)	2.96 (402.68)	1.54 (1.36)	2.90 (2.60)
	Respiratory tract infection	318	2.91 (2.60–3.25)	2.90 (385.03)	1.51 (1.33	2.85 (2.55)
	Oral candidiasis	317	6.78 (6.05–7.60)	6.76 (1461.78)	2.68 (2.48)	6.41 (5.72)
	Viral infection	315	2.39 (2.13–2.67)	2.38 (247.05)	1.23 (1.06)	2.35 (2.10)
	Pharyngitis streptococcal	268	6.55 (5.79–7.41)	6.53 (1181.49)	2.63 (2.42)	6.20 (5.48)
	Kidney infection^a^	250	2.91 (2.57–3.30)	2.91 (304.03)	1.51 (1.31)	2.85 (2.52)
	Pharyngitis	226	4.34 (3.80–4.96)	4.33 (555.87)	2.07 (1.85)	4.20 (3.67)
	Oral herpes	224	2.86 (2.50–3.26)	2.85 (262.36)	1.49 (1.27)	2.80 (2.45)
	Tuberculosis	188	3.70 (3.20–4.28)	3.69 (356.55)	1.85 (1.61)	3.60 (3.11)
	Tonsillitis	173	8.08 (6.92–9.44)	8.07 (994.01)	2.92 (2.63)	7.56 (6.47)
	Tooth abscess	169	3.95 (3.39–4.60)	3.94 (357.59)	1.94 (1.68)	3.83 (3.29)
	Eye infection	160	3.50 (2.99–4.09)	3.49 (275.38)	1.77 (1.51)	3.41 (2.91)
	Skin infection	142	3.03 (2.56–3.58)	3.02 (186.78)	1.57 (1.29)	2.96 (2.51)
	Erysipelas	142	6.65 (5.61–7.88)	6.64 (639.16)	2.65 (2.34)	6.30 (5.31)
	Tooth infection	141	2.61 (2.21–3.08)	2.61 (136.21)	1.36 (1.09)	2.57 (2.17)
	Rhinitis	135	4.12 (3.47–4.89)	4.11 (305.82)	2.00 (1.70)	3.99 (3.36)
	Furuncle	129	3.85 (3.23–4.59)	3.85 (262.10)	1.90 (1.60)	3.74 (3.14)
	Coronavirus infection	119	3.43 (2.86–4.12)	3.43 (198.47)	1.75 (1.44)	3.35 (2.79)
	Laryngitis	114	2.70 (2.24–3.25)	2.70 (118.93)	1.41 (1.10)	2.66 (2.21)
Investigations	C-reactive protein increased	357	2.68 (2.42–2.98)	2.68 (366.02)	1.40 (1.23)	2.63 (2.37)
	SARS-CoV-2 test positive	151	4.29 (3.65–5.05)	4.29 (365.29)	2.05 (1.78)	4.15 (3.53)
Musculoskeletal and connective tissue disorders	Arthralgia^a^	5,682	3.63 (3.53–3.73)	3.46 (9788.63)	1.76 (1.72)	3.38 (3.29)
	Psoriatic arthropathy	2,541	19.96 (19.12–20.84)	19.42 (37410.46)	4.04 (3.97)	16.50 (15.80)
	Arthritis	1,672	5.37 (5.11–5.65)	5.29 (5554.55)	2.35 (2.27)	5.08 (4.84)
	Musculoskeletal stiffness^a^	1,381	3.82 (3.61–4.03)	3.77 (2725.03)	1.88 (1.79)	3.67 (3.48)
	Joint swelling	1,280	2.65 (2.50–2.80)	2.62 (1261.42)	1.37 (1.28)	2.58 (2.44)
	Ankylosing spondylitis	724	18.44 (17.04–19.96)	18.30 (10057.30)	3.97 (3.83)	15.69 (14.49)
	Neck pain	597	2.60 (2.39–2.82)	2.59 (568.61)	1.35 (1.22)	2.55 (2.35)
	Joint stiffness^a^	390	3.67 (3.32–4.06)	3.66 (727.79)	1.83 (1.67)	3.57 (3.22)
	Spinal pain^a^	296	5.19 (4.62–5.84)	5.18 (950.43)	2.32 (2.12)	4.98 (4.43)
	Fibromyalgia^a^	222	2.43 (2.12–2.77)	2.42 (181.36)	1.26 (1.05)	2.39 (2.09)
	Musculoskeletal discomfort^a^	186	2.46 (2.12–2.84)	2.45 (156.77)	1.28 (1.04)	2.42 (2.09)
Nervous system disorders	Movement disorder^a^	315	2.82 (2.52–3.15)	2.81 (357.80)	1.47 (1.29)	2.76 (2.47)
	Hypokinesia^a^	191	2.82 (2.44–3.26)	2.82 (218.30)	1.47 (1.24)	2.77 (2.40)
	Anosmia^a^	135	3.41 (2.87–4.05)	3.41 (222.55)	1.74 (1.45)	3.33 (2.81)
Psychiatric disorders	Stress^a^	869	3.08 (2.88–3.30)	3.06 (1176.79)	1.59 (1.48)	3.00 (2.81)
Respiratory, thoracic and mediastinal disorders	Oropharyngeal pain	1,393	3.61 (3.42–3.81)	3.57 (2499.39)	1.80 (1.72)	3.48 (3.30)
	Rhinorrhoea	782	2.83 (2.63–3.04)	2.81 (890.59)	1.47 (1.36)	2.76 (2.57)
	Nasal congestion	621	2.62 (2.42–2.84)	2.61 (603.20)	1.36 (1.24)	2.57 (2.37)
Skin and subcutaneous tissue disorders	Psoriasis	1,2746	31.09 (30.45–31.74)	26.79 (252731.11)	4.42 (4.39)	21.46 (21.02)
	Pruritus	4,193	2.86 (2.77–2.95)	2.77 (4715.88)	1.45 (1.40)	2.73 (2.64)
	Skin exfoliation	1,270	3.14 (2.97–3.33)	3.11 (1776.38)	1.61 (1.52)	3.05 (2.88)
	Skin plaque	960	35.76 (33.22–38.49)	35.38 (23872.01)	4.73 (4.59)	26.58 (24.69)
	Skin lesion	716	6.92 (6.41–7.46)	6.87 (3370.28)	2.70 (2.58)	6.50 (6.03)
	Skin disorder	568	4.41 (4.05–4.79)	4.38 (1425.38)	2.09 (1.95)	4.25 (3.90)
	Blister	565	2.56 (2.35–2.78)	2.55 (519.26)	1.33 (1.20)	2.51 (2.31)
	Macule	519	53.62 (48.22–59.63)	53.31 (17547.72)	5.15 (4.91)	35.45 (31.88)
	Skin fissures	518	7.87 (7.20–8.61)	7.83 (2871.83)	2.88 (2.73)	7.35 (6.72)
	Skin haemorrhage	497	9.69 (8.84–10.62)	9.64 (3521.20)	3.15 (2.99)	8.90 (8.12)
	Skin discolouration	473	2.32 (2.12–2.54)	2.31 (345.37)	1.19 (1.05)	2.28 (2.08)
	Scab	226	4.51 (3.95–5.16)	4.50 (590.67)	2.12 (1.90)	4.36 (3.81)
	Pigmentation disorder^a^	174	7.28 (6.24–8.49)	7.27 (878.61)	2.78 (2.50)	6.85 (5.88)
	Skin mass	132	4.35 (3.65–5.17)	4.34 (325.90)	2.07 (1.77)	4.21 (3.53)
	Pustular psoriasis	128	11.64 (9.70–13.98)	11.63 (1117.12)	3.40 (3.02)	10.55 (8.78)
	Nail disorder^a^	110	3.45 (2.85–4.17)	3.45 (184.79)	1.75 (1.43)	3.37 (2.78)

a Emerging findings of secukinumab associated AEs from FAERS database.

ROR, reporting odds ratio; CI, confidence interval; PRR, proportional reporting ratio; χ^2^, chi-squared; IC, information component; EBGM, empirical Bayesian geometric mean.

Due to all the medical and health-related PTs were collected by FAERS, it was worth noting that we also found some signals unrelated to the drug (Supplementary Table S3), which mainly focused on injury, poisoning and procedural complications (SOC: 10022117), product issues (SOC: 10077536), social circumstances (SOC: 10041244) and surgical and medical procedures (SOC: 10042613).

### Time to Onset of Secukinumab-Associated Adverse Events

**FIGURE 2:**
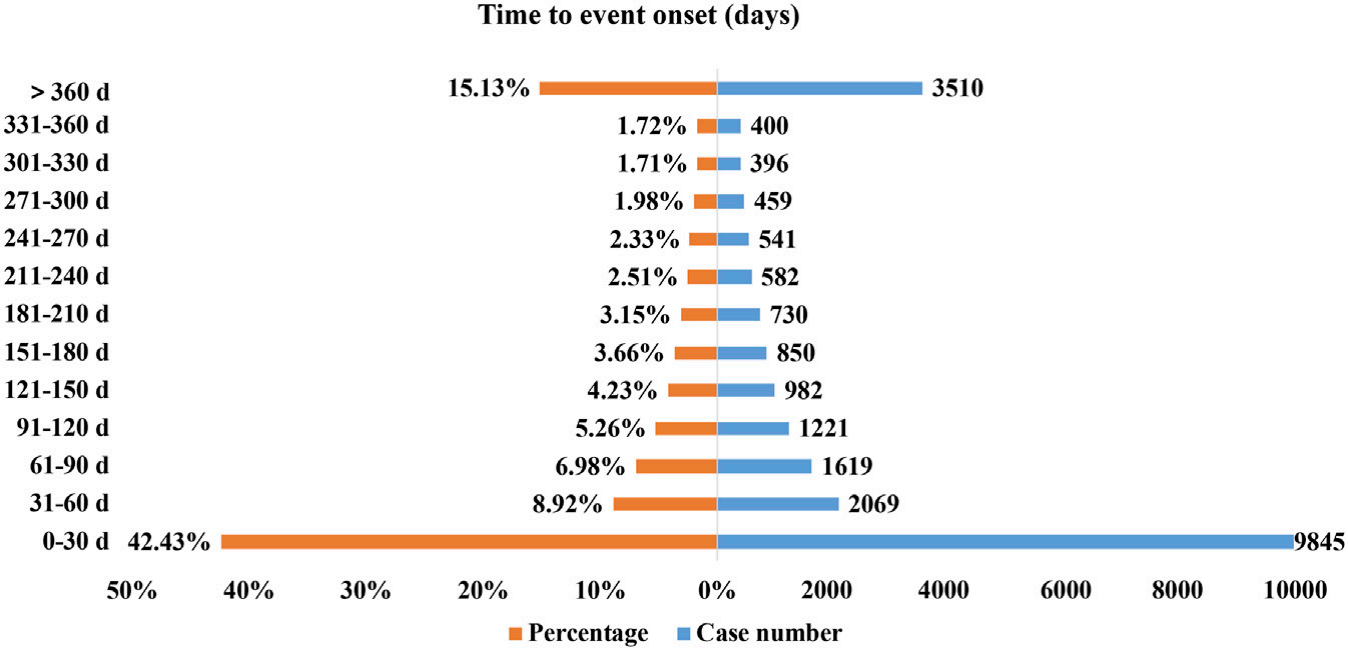
Time to onset of secukinumab-related AEs.

Excluding inaccurate, missing or unknown onset time reports, a total of 23204 AEs reported onset time and the median time to onset was 56 days (interquartile range [IQR] 5–214 days). As Figure 2 illustrated, most of the onsets of secukinumab occurred within the first 1 (*n* = 9845, 42.43%), 2 (*n* = 2069, 8.92%), 3 (*n* = 1619, 6.98%) and 4 months (*n* = 1221, 5.26%) after initiation of secukinumab. About 15.13% (*n* = 3510) of AEs occurred 1 year later.

## Discussion

To the best of our knowledge, this is the first most comprehensive and systematic pharmacovigilance study on secukinumab-associated AEs by post-marketing based on the FAERS database. We presented a more accurate and detailed description and characterization of secukinumab-associated AEs to date. Three years after the approval of secukinumab, reports of AEs since 2018 have continued to increase (averaging over 17,000 cases per year) due to the widespread use and increased awareness of healthcare professionals, strongly underling the need for constant epidemiological surveillance. In our study, secukinumab demonstrated a higher AEs proportion in patients aged 18–65 years old (30.88%) with the average age of 54 possibly because of the fact that the safety and efficacy of secukinumab in children under 18 years old were not well established. In addition, the AEs signals detected appeared to mainly driven by a greater risk in those over age 50 years.

The most common and significant SOCs such as infections and infestations, respiratory, thoracic and mediastinal disorders, skin and subcutaneous tissue disorders, immune system disorders, and ear and labyrinth disorders were consistent with the safety data in the label and clinical trials (McInnes et al., 2015; Deodhar et al., 2019). Among the SOC of infections and infestations, AEs with the highest number of reports were nasopharyngitis, influenza, sinusitis, and respiratory tract infection. By integrating pooled clinical trial and post-marketing surveillance data, A. Deodhar et al. (2019) found that the exposure-adjusted incidence rates (EAIRs) for serious infections reached 1.4, 1.9 and 1.2 per 100 patient-years in secukinumab treatment of PsO, PsA and AS, respectively. Upper respiratory tract infection was the most common type of infection and other infections like esophageal and gastrointestinal candidiasis also included, which was in line with our findings. Multiple meta-analysis studies of the safety also showed that the most common AEs associated with the use of secukinumab were nasopharyngitis, headache, upper respiratory tract infection, diarrhea, arthralgia and pruritus (Ryoo et al., 2016; Bai et al., 2019). It is possible that IL-17A attracts myeloid cells to infected tissues when it induces some types of chemokines, including CXCL1, CXCL2, and IL-8 (McGeachy et al., 2019). Thus, IL-17A would recruit neutrophils during host defense against extracellular bacterial and fungal pathogens, promoting production of antibacterial molecules and acute phase proteins (Song et al., 2011; Loricera et al., 2021). Bacterial and *candida* infections are expected when IL-17 pathway is blocked by secukinumab. On the other hand, the frequency of these AEs demonstrated that secukinumab might suppress the immune response as evidenced by our study that decreased immune responsiveness was detected as significant signal strength being ROR 12.62 (11.49–13.85), PRR 12.55 (4711.97), IC 3.50 (3.33), EBGM 11.30 (10.29), respectively.

It was noteworthy that the long-term use of secukinumab was associated with a risk of dermatic AEs, and the most common AEs such as pruritus, skin exfoliation, and blister were detected in skin and subcutaneous tissue disorders. Several studies had also reported infective dermatitis, atopic dermatitis eczema-like and dyshidrotic eczema after treatment with secukinumab (Burlando et al., 2019; Bose and Beecker, 2020; Utiyama et al., 2022). The reason why dermatitis and eczema developed during secukinumab therapy might be attributed to that psoriasis and eczema were considered to be diseases caused by an imbalance in the Th1/Th2 immune response, with Th1 being more prominent in psoriasis and Th2 predominating in dermatitis and eczema (Eyerich et al., 2011; Burlando et al., 2019). Secukinumab can block Th1 pathway, and in turn Th2 balance will increase, inducing dermatitis and eczema. Consistent with our results, pharmacovigilance risk assessment committee (PRAC) put forward evaluation opinions on the safety signals of secukinumab through data mining of EudraVigilance database and literature reports. Based on this, the European Medicines Agency (EMA) also revised the instructions for secukinumab in 2019, adding exfoliative dermatitis to the list of adverse reactions. Then, in 2020, the Pharmaceuticals and Medical Device Agency (PMDA) in Japan made the same revision.

Studies using secukinumab for the treatment of PsO, PsA and AS, have previously reported both exacerbations and new-onset cases of inflammatory bowel disease (IBD) (Hueber et al., 2012; Fobelo Lozano et al., 2018). Similarly, IBD and irritable bowel syndrome (all values well above the threshold for significance) identified as the most common gastrointestinal AEs caused by secukinumab in our analysis were estimated to have a strong risk signal, but a relatively low frequency of occurrence in the clinical trials. Consistently, Stefan Schreiber et al. (Schreiber et al., 2019) retrospectively analyzed the safety of secukinumab for treatment of PsO, PsA, and AS in 21 clinical trials and found that the incidence rate of IBD caused by secukinumab was 0.56%. In general, IL-17A drives pathogenic inflammation in PsO. However, IL-17A has also been found to play a vital role in gastrointestinal homeostasis and tissue repair (O'Connor et al., 2009). IL-17A inhibition may have dual effects, alleviating inflammation, but also damaging the function of already impaired intestinal epithelial barrier, causing colitis (Ogawa et al., 2004; Schreiber et al., 2019). Hence, there is an implication for clinicians that patients should be screened for IBD or a family history of IBD before clinical use, and the symptoms and signs of IBD should also be closely monitored. In 2016, it was added to section of warnings and cautions when FDA revised the instructions for secukinumab.

In SOC of ear disorders, in addition to external ear inflammation and middle ear inflammation that presented in the drug label, new PT signals of otorrhoea and tympanic membrane perforation, which were not observed in clinical trials, were detected in our analysis, suggesting clinicians should pay more attention to it. Uveitis is a common AE of the eye disorders induced by secukinumab. In a cohort of 3616 spondyloarthritis patients treated with secukinumab or TNF-α inhibitors, the new-onset anterior uveitis occurred in only 1% of patients (Lindström et al., 2021). However, there were differences among the drugs, with adalimumab being the lowest at 0.5% and secukinumab (1.3%), certolizumab (1.6%) and etanercept (1.2%), but the frequency of occurrence was too low to draw any reliable conclusions (Lindström et al., 2021). In another analysis that pooled data from randomized controlled trials of secukinumab for ankylosing spondylitis, 1.5% of patients developed new-onset uveitis (Deodhar et al., 2020). In our study, uveitis was reported in 281 cases with significant AE signals, further strengthening the credibility of the clinical trial results.

Compared with a meta-analysis of 43 studies, which provided real-world evidence of secukinumab in PsO treatment, indicating that AEs were consistent with rates observed in clinical trials with no new or unexpected safety signals in duration from 3 to 24 months (Augustin et al., 2020), our study raised different safety concerns and suggested that secukinumab had the potential for unexpected and new significant AEs at SOC levels, such as general disorders and administration site conditions (SOC: 10018065, *n* = 40825), musculoskeletal and connective tissue disorders (SOC: 10040785, *n* = 30047). As high-molecular-weight biologics, the majority of secukinumab were injected subcutaneously, with few being given intravenously. The injection site adverse reactions were frequently associated with pain, erythema, hemorrhage and edema (Murdaca et al., 2013). Significant AEs signals of injection site pain, vessel puncture site haemorrhage and injection site nerve damage were examined in our study, corresponding to Elsie Grace’s study, who reported injection site adverse reactions caused by biologicals for the treatment of psoriasis through a pharmacovigilance study based on FAERS database with no onset time (Grace et al., 2020). Injection site pain was the most common AEs, which were reported in 20% ixekizumab and 25% secukinumab of all injection site adverse reactions (Grace et al., 2020). The main indications for secukinumab are patients with PsO, PsA, and AS, and the arthritis, psoriatic arthropathy, and ankylosing spondylitis AEs signals in this study might be considered to be due to disease progression or reduced efficacy of biologics. Sometimes, the curative effect of biological agents in clinical application maybe weakened to varying degrees during the treatment (Tsakok et al., 2021). Nevertheless, some AEs of musculoskeletal and connective tissue disorders, like arthralgia, musculoskeletal stiffness, joint stiffness and fibromyalgia might be thought to be associated with secukinumab treatment. However, the exact induction mechanisms remained unclear.

Some other unexpected and new significant AEs signals, which were not reported by regulatory trials, such as bone marrow oedema, hypokinesia, anosmia, Bell’s palsy, aortic elongation, parotid gland enlargement, angular cheilitis and stress were detected in our analysis. More importantly, serious AEs, such as cutaneous T-cell lymphoma and acral lentiginous melanoma, were also detected with significant signals strength. Several clinical studies also reported malignant or unspecified tumors by secukinumab treatment (Baraliakos et al., 2019; Braun et al., 2019; Pavelka et al., 2020). Physicians should be alert to these unexpected and serious AEs, and therefore monitor patients for extended periods of time after secukinumab administration, and FDA may revise and give warnings on the label if necessary.

Although AEs of hypercholesterolaemia, neutrophil count decreased and transaminases increased have been reported in some clinical trials and specifications, no significant signals of these AEs were detected in our data analysis, consistent with a report of post-marketing safety data of secukinumab based on a total of 5181, 1380 and 794 patients from PsO, PsA and AS clinical trials (Deodhar et al., 2019; Loricera et al., 2021). With large-sample cases in FAERS database, it is suggested that the reports proportion of these AEs may be low in the large population, which provides substantial evidence for the revision of the specification of secukinumab in the future.

Clinical trials have shown that oral candidiasis and erysipelas typically appeared in the first 16 weeks of secukinumab treatment, while tuberculosis occurred 2 months after the last dose (Kivitz et al., 2018; Braun et al., 2019). In the present study, the median time to onset was 56 days (interquartile range [IQR] 5–214 days), and most of the AEs occurred within the first 1 (*n* = 9845, 42.43%), 2 (*n* = 2069, 8.92%), 3 (*n* = 1619, 6.98%) and 4 months (*n* = 1221, 5.26%) after initiation of secukinumab, corresponding to previous clinical trials data. Hence, a more precise definition of the timing of onset and early recognition of already known AEs deserves clear attention from both patients and clinicians, which could help clinicians take effective measures to prevent, reduce and identify AEs in advance or in time.

At present, there are few real-world large-sample safety studies about secukinumab based on FAERS database, with only one article been searched in PubMed, focusing on injection site reactions (Grace et al., 2020). Other safety researches on secukinumab were meta-analyses or short-term clinical trials with limited sample size, follow-up time and observable AEs. Moreover, the time to onset of AEs was unknown. Excitingly, our study obtained the largest number of secukinumab cases with 89,228 reports and 254,886 AEs to date. In addition to adverse reactions that are consistent with drug specifications and clinical trials, we have identified a large number of new and unexpected significant AEs. Moreover, the onset time of AEs and the serious outcomes of AEs, including severity and proportion are also analyzed, in order to provide a comprehensive and valuable reference for the safety study of secukinumab.

Despite the advantages of real-world large-sample research and the data mining techniques in this study, there are still some limitations that warrant discussion. First, FAERS is a spontaneous reporting system with incomplete and incorrect information collected from different countries and professionals, thus the quality might be variable, which may lead to bias in the analysis. It is difficult to control confounders such as dosage, duration of use, comorbidities, drug combinations, and other factors that may influence AEs. Second, the incidence rate of each AEs can’t be calculated because of lacking total numbers of patients receiving secukinumab treatment. Third, this study fails to confirm the causal relationship between the target drug and AEs, because disproportionality analysis only provided an evaluation of the signal strength, which was only statistically significant, neither quantified risk nor existed causality. Further experimental studies are still needed to validate the results. Despite these limitations, our results would provide a valuable reference for healthcare professionals to closely follow-up patients and monitor the associated adverse reactions of secukinumab.

## Conclusion

Our pharmacovigilance analysis explored reports of secukinumab-associated AEs in the FAERS database. 89,228 reports of secukinumab as the PS and 254,886 AEs induced by secukinumab were identified. Common AEs in SOC levels, such as infestations, respiratory tract infection, immune system disorders, eye and ear disorders, skin and subcutaneous tissue disorders and gastrointestinal disorders should be highly concerned. Unexpected and new significant AEs as vessel puncture site haemorrhage, injection site nerve damage, hypokinesia, anosmia, musculoskeletal stiffness and malignant or unspecified tumors might also occur. This long-term post-marketing safety evaluation provides a broader understanding of secukinumab’s safety and supports its safe and rational use in chronic systemic inflammatory diseases. Further studies are still needed to address the mechanisms underlying unexpected and new AEs associated with secukinumab and assess the causality of the cases to draw conclusions on the strength of the relationships.

## Data Availability

The original contributions presented in the study are included in the article/Supplementary Material; further inquiries can be directed to the corresponding author.
