# Supplementary material for: Post-Marketing Safety Concerns With Secukinumab: A Disproportionality Analysis of the FDA Adverse Event Reporting System
Source: Front Pharmacol. 2022 Jun 8;13:862508. doi: 10.3389/fphar.2022.862508 (PMC9214234; doi:10.3389/fphar.2022.862508)
Supplement: Supplementary file 1 [file DataSheet1.docx]

**Supplementary Table 1. Four major algorithms used for signal detection.**

| Algorithms | Equation | Criteria |
| --- | --- | --- |
| ROR | ROR=ad/b/c | lower limit of 95% CI>1, N≥3 |
|  | 95%CI=e^ln(ROR)±1.96(1/a+1/b+1/c+1/d)^0.5^ |  |
| PRR | PRR=a(c+d)/c/(a+b) | PRR≥2, χ^2^≥4, N≥3 |
|  | χ^2^=[(ad-bc)^2](a+b+c+d)/[(a+b)(c+d)(a+c)(b+d)] |  |
| BCPNN | IC=log_2_a(a+b+c+d)(a+c)(a+b) | IC025>0 |
|  | 95%CI= E(IC) ± 2V(IC)^0.5 |  |
| MGPS | EBGM=a(a+b+c+d)/(a+c)/(a+b) | EBGM05>2 |
|  | 95%CI=e^ln(EBGM)±1.96(1/a+1/b+1/c+1/d)^0.5^ |  |

Equation: a, number of reports containing both the target drug and target adverse drug reaction; b, number of reports containing other adverse drug reaction of the target drug; c, number of reports containing the target adverse drug reaction of other drugs; d, number of reports containing other drugs and other adverse drug reactions. 95%CI, 95% confidence interval; N, the number of reports; χ^2^, chi-squared; IC, information component; IC025, the lower limit of 95% CI of the IC; E(IC), the IC expectations; V(IC), the variance of IC; EBGM, empirical Bayesian geometric mean; EBGM05, the lower limit of 95% CI of EBGM.

**Supplementary Table 2. Signal strength of reports of secukinumab at the Preferred Term (PT) level in FAERS database.**

| SOC | Preferred Terms (PTs) | Secukinumab Cases  Reporting PT | ROR  (95% two-sided CI) | PRR (χ2) | IC (IC025) | EBGM (EBGM05) |
| --- | --- | --- | --- | --- | --- | --- |
| Blood and lymphatic system disorders | Bone marrow oedema* | 15 | 3.84（2.29-6.43） | 3.84（30.33） | 1.90（0.82） | 3.73（2.23） |
| Congenital, familial and genetic disorders | Dolichocolon | 4 | 13.27（4.68-37.59） | 13.27（40.19） | 3.57（0.06） | 11.87（4.19） |
| Ear and labyrinth disorders | Ear disorder | 56 | 2.77（2.13-3.62） | 2.77（61.74） | 1.45（0.99） | 2.72（2.09） |
|  | Otorrhoea* | 38 | 5.16（3.73-7.15） | 5.16（121.42） | 2.31（1.65） | 4.96（3.58） |
|  | Tympanic membrane perforation* | 22 | 3.21（2.10-4.91） | 3.21（32.44） | 1.65（0.83） | 3.14（2.06） |
|  | Excessive cerumen production | 15 | 4.43（2.64-7.43） | 4.43（38.22） | 2.10（0.98） | 4.29（2.56） |
|  | External ear inflammation | 8 | 13.27（6.35-27.71） | 13.27（80.38） | 3.57（1.18） | 11.87（5.68） |
|  | Middle ear inflammation | 5 | 11.69（4.63-29.47） | 11.68（43.87） | 3.41（0.41） | 10.59（4.20） |
| Eye disorders | Iritis | 43 | 3.75（2.77-5.09） | 3.75（83.81） | 1.87（1.30） | 3.66（2.70） |
|  | Iridocyclitis | 43 | 3.60（2.66-4.89） | 3.60（78.16） | 1.81（1.25） | 3.52（2.59） |
| Gastrointestinal disorders | Angular cheilitis | 17 | 4.67（2.87-7.60） | 4.67（46.96） | 2.17（1.12） | 4.51（2.78） |
|  | Coating in mouth | 14 | 4.54（2.66-7.76） | 4.54（37.03） | 2.13（0.95） | 4.39（2.57） |
|  | Parotid gland enlargement* | 11 | 5.17（2.82-9.47） | 5.16（35.18） | 2.31（0.88） | 4.97（2.71） |
|  | Enlarged uvula* | 11 | 4.38（2.40-8.02） | 4.38（27.56） | 2.09（0.73） | 4.25（2.32） |
|  | Anal ulcer | 11 | 3.78（2.07-6.91） | 3.78（21.72） | 1.88（0.58） | 3.68（2.02） |
|  | Plicated tongue* | 8 | 4.40（2.17-8.93） | 4.40（20.15） | 2.09（0.43） | 4.26（2.10） |
|  | Oral lichen planus | 8 | 4.31（2.12-8.74） | 4.31（19.50） | 2.06（0.42） | 4.17（2.06） |
|  | Cryptitis | 5 | 10.08（4.02-25.26） | 10.08（37.25） | 3.21（0.35） | 9.27（3.70） |
| General disorders and administration site conditions | Therapeutic response delayed* | 36 | 3.36（2.41-4.68） | 3.36（57.77） | 1.72（1.10） | 3.28（2.36） |
|  | Breakthrough pain* | 35 | 4.00（2.86-5.61） | 4.00（75.91） | 1.96（1.31） | 3.89（2.78） |
|  | Discharge* | 23 | 4.38（2.89-6.65） | 4.38（57.54） | 2.08（1.23） | 4.24（2.79） |
|  | Inflammatory pain* | 21 | 8.60（5.51-13.43） | 8.60（130.21） | 3.00（1.88） | 8.02（5.14） |
|  | Vessel puncture site haemorrhage* | 7 | 6.00（2.80-12.85） | 6.00（27.55） | 2.52（0.53） | 5.72（2.67） |
|  | Injection site nerve damage* | 6 | 5.71（2.51-13.00） | 5.71（22.10） | 2.45（0.31） | 5.46（2.40） |
| Hepatobiliary disorders | Alcoholic liver disease | 8 | 6.74（3.30-13.79） | 6.74（36.72） | 2.68（0.78） | 6.39（3.12） |
| Immune system disorders | Immunosuppression | 99 | 2.91（2.39-3.56） | 2.91（120.83） | 1.52（1.18） | 2.86（2.34） |
|  | Rubber sensitivity | 41 | 5.99（4.37-8.21） | 5.99（161.01） | 2.51（1.86） | 5.71（4.17） |
| Infections and infestations | Rash pustular | 91 | 3.05（2.47-3.75） | 3.04（121.27） | 1.58（1.22） | 2.98（2.42） |
|  | Oesophageal candidiasis | 89 | 6.39（5.16-7.91） | 6.38（380.39） | 2.60（2.19） | 6.07（4.90） |
|  | Viral upper respiratory tract infection | 86 | 2.54（2.05-3.14） | 2.54（78.11） | 1.32（0.97） | 2.50（2.02） |
|  | Folliculitis | 83 | 3.97（3.19-4.95） | 3.97（177.57） | 1.95（1.56） | 3.86（3.10） |
|  | Streptococcal infection | 75 | 3.99（3.17-5.03） | 3.99（161.65） | 1.95（1.54） | 3.88（3.08） |
|  | Suspected COVID-19* | 71 | 4.34（3.42-5.50） | 4.34（174.85） | 2.07（1.64） | 4.20（3.31） |
|  | Tinea pedis | 68 | 13.01（10.11-16.74） | 13.00（668.55） | 3.54（2.95） | 11.65（9.05） |
|  | Abscess limb | 60 | 3.29（2.55-4.26） | 3.29（92.81） | 1.69（1.23） | 3.22（2.49） |
|  | Arthritis infective | 59 | 2.97（2.3-3.85） | 2.97（75.07） | 1.54（1.09） | 2.92（2.25） |
|  | Oral infection | 47 | 3.50（2.62-4.68） | 3.50（81.06） | 1.77（1.24） | 3.41（2.55） |
|  | Oral fungal infection | 46 | 4.27（3.18-5.74） | 4.27（110.73） | 2.05（1.49） | 4.14（3.08） |
|  | Vulvovaginal candidiasis | 44 | 8.64（6.35-11.75） | 8.63（274.01） | 3.01（2.31） | 8.04（5.91） |
|  | Latent tuberculosis | 44 | 4.66（3.44-6.30） | 4.65（120.80） | 2.17（1.58） | 4.50（3.32） |
|  | Infection susceptibility increased | 44 | 4.34（3.21-5.87） | 4.34（108.45） | 2.07（1.50） | 4.20（3.11） |
|  | Pustule | 41 | 6.72（4.90-9.21） | 6.71（187.14） | 2.67（2.00） | 6.36（4.64） |
|  | Impetigo | 37 | 6.87（4.92-9.58） | 6.87（173.88） | 2.70（1.98） | 6.50（4.66） |
|  | Labyrinthitis | 33 | 3.32（2.35-4.70） | 3.32（51.84） | 1.70（1.05） | 3.25（2.30） |
|  | Staphylococcal skin infection | 28 | 6.04（4.12-8.84） | 6.04（111.13） | 2.53（1.70） | 5.76（3.93） |
|  | Dengue fever | 28 | 3.03（2.08-4.42） | 3.03（37.09） | 1.57（0.87） | 2.98（2.04） |
|  | Otitis externa | 27 | 5.97（4.05-8.80） | 5.97（105.60） | 2.51（1.66） | 5.70（3.87） |
|  | Nail infection | 26 | 4.15（2.81-6.15） | 4.15（59.79） | 2.01（1.23） | 4.03（2.72） |
|  | Oropharyngeal candidiasis | 25 | 6.63（4.42-9.93） | 6.63（112.19） | 2.65（1.73） | 6.28（4.19） |
|  | Lower respiratory tract infection viral | 21 | 8.18（5.25-12.76） | 8.18（122.59） | 2.94（1.84） | 7.65（4.91） |
|  | Genital candidiasis | 21 | 31.76（19.47-51.81） | 31.76（477.93） | 4.61（2.80） | 24.50（15.02） |
|  | Bursitis infective* | 20 | 8.54（5.41-13.47） | 8.53（122.82） | 2.99（1.84） | 7.96（5.04） |
|  | Ear infection fungal | 19 | 14.58（9.02-23.58） | 14.58（210.48） | 3.69（2.24） | 12.89（7.97） |
|  | Oesophageal infection | 18 | 11.29（6.94-18.36） | 11.29（152.05） | 3.36（2.00） | 10.27（6.31） |
|  | Fungal oesophagitis | 18 | 7.32（4.54-11.80） | 7.32（91.63） | 2.79（1.62） | 6.90（4.28） |
|  | Skin candida | 16 | 9.19（5.51-15.33） | 9.19（107.22） | 3.09（1.72） | 8.52（5.11） |
|  | Pulpitis dental | 16 | 4.22（2.56-6.96） | 4.22（37.74） | 2.03（0.97） | 4.09（2.48） |
|  | Peritonsillar abscess | 14 | 4.25（2.49-7.25） | 4.25（33.37） | 2.04（0.88） | 4.12（2.41） |
|  | Campylobacter gastroenteritis | 14 | 5.50（3.21-9.41） | 5.49（48.86） | 2.40（1.15） | 5.27（3.08） |
|  | Tinea cruris | 13 | 6.79（3.87-11.90） | 6.79（60.16） | 2.68（1.28） | 6.43（3.67） |
|  | Skin bacterial infection | 13 | 4.71（2.70-8.21） | 4.71（36.29） | 2.18（0.93） | 4.54（2.61） |
|  | Gastrointestinal fungal infection | 13 | 6.75（3.85-11.83） | 6.75（59.77） | 2.68（1.28） | 6.40（3.65） |
|  | Gingival abscess | 12 | 3.87（2.17-6.88） | 3.87（24.59） | 1.91（0.67） | 3.76（2.12） |
|  | Fungal pharyngitis | 12 | 9.87（5.46-17.85） | 9.87（87.29） | 3.18（1.50） | 9.09（5.03） |
|  | Groin infection | 11 | 5.03（2.74-9.21） | 5.03（33.83） | 2.27（0.86） | 4.84（2.64） |
|  | Cellulitis staphylococcal | 11 | 4.14（2.27-7.57） | 4.14（25.21） | 2.01（0.67） | 4.02（2.20） |
|  | Tinea versicolour | 10 | 7.67（4.04-14.59） | 7.67（54.01） | 2.85（1.12） | 7.21（3.79） |
|  | Staphylococcal abscess | 10 | 4.97（2.63-9.37） | 4.97（30.23） | 2.26（0.76） | 4.78（2.54） |
|  | Tonsillitis bacterial | 9 | 10.28（5.18-20.41） | 10.28（68.56） | 3.24（1.20） | 9.44（4.76） |
|  | Mumps* | 9 | 6.86（3.49-13.46） | 6.86（42.20） | 2.70（0.92） | 6.49（3.3） |
|  | Genital abscess | 9 | 6.05（3.09-11.85） | 6.05（35.82） | 2.53（0.83） | 5.77（2.94） |
|  | Pharyngitis bacterial | 8 | 7.69（3.75-15.77） | 7.69（43.31） | 2.85（0.87） | 7.22（3.52） |
|  | Nasal abscess | 8 | 9.24（4.48-19.06） | 9.24（53.96） | 3.10（0.99） | 8.56（4.15） |
|  | Mucocutaneous candidiasis | 8 | 30.47（13.84-67.07） | 30.47（175.89） | 4.57（1.45） | 23.73（10.78） |
|  | Infected dermal cyst | 8 | 4.55（2.24-9.23） | 4.54（21.18） | 2.14（0.46） | 4.39（2.16） |
|  | Gastrointestinal candidiasis | 8 | 5.75（2.82-11.73） | 5.75（29.75） | 2.46（0.66） | 5.50（2.70） |
|  | Borrelia infection | 8 | 6.09（2.99-12.44） | 6.09（32.16） | 2.54（0.70） | 5.81（2.85） |
|  | Oral pustule | 7 | 6.99（3.25-15.03） | 6.99（33.63） | 2.72（0.64） | 6.61（3.07） |
|  | Leprosy* | 7 | 8.27（3.83-17.87） | 8.27（41.43） | 2.95（0.75） | 7.73（3.58） |
|  | Balanitis candida | 7 | 11.25（5.15-24.54） | 11.25（58.91） | 3.36（0.91） | 10.24（4.69） |
|  | Ear infection staphylococcal | 6 | 11.87（5.10-27.62） | 11.86（53.52） | 3.43（0.71） | 10.74（4.61） |
|  | Conjunctivitis viral | 6 | 5.77（2.53-13.12） | 5.77（22.38） | 2.46（0.31） | 5.51（2.42） |
|  | Abscess sweat gland | 6 | 44.07（16.94-114.69） | 44.07（176.78） | 4.96（0.99） | 31.15（11.97） |
|  | Tonsillitis streptococcal | 5 | 7.56（3.05-18.75） | 7.56（26.52） | 2.83（0.22） | 7.11（2.87） |
|  | Oral bacterial infection | 5 | 7.04（2.85-17.43） | 7.04（24.26） | 2.73（0.18） | 6.66（2.69） |
|  | Lymphadenitis bacterial | 5 | 7.24（2.92-17.93） | 7.24（25.13） | 2.77（0.20） | 6.83（2.76） |
|  | Ear lobe infection | 5 | 11.96（4.74-30.19） | 11.96（44.97） | 3.43（0.42） | 10.82（4.28） |
|  | Fungal rhinitis | 4 | 11.43（4.07-32.10） | 11.43（34.25） | 3.38（0.02） | 10.38（3.70） |
|  | Bronchitis mycoplasmal | 4 | 68.55（19.34-242.94） | 68.55（159.76） | 5.38（0.16） | 41.53（11.72） |
| Investigations | Grip strength decreased* | 87 | 2.97（2.40-3.68） | 2.97（110.39） | 1.54（1.18） | 2.91（2.35） |
|  | Mycobacterium tuberculosis complex test positive | 54 | 4.56（3.47-5.99） | 4.56（143.49） | 2.14（1.62） | 4.40（3.35） |
|  | Tuberculin test positive | 41 | 3.66（2.68-4.99） | 3.66（76.42） | 1.83（1.26） | 3.57（2.61） |
|  | Coronavirus test positive* | 33 | 6.66（4.68-9.46） | 6.65（148.9） | 2.66（1.89） | 6.31（4.44） |
|  | Swollen joint count increased* | 15 | 5.30（3.15-8.91） | 5.30（49.77） | 2.35（1.16） | 5.09（3.03） |
|  | Cell marker increased* | 15 | 6.20（3.68-10.43） | 6.19（61.63） | 2.56（1.32） | 5.90（3.50） |
|  | Antinuclear antibody increased | 14 | 4.10（2.40-7.00） | 4.10（31.57） | 1.99（0.85） | 3.98（2.33） |
|  | Psoriasis area severity index increased | 13 | 26.74（14.53-49.22） | 26.73（255.59） | 4.42（2.14） | 21.42（11.64） |
|  | Occult blood | 11 | 4.71（2.58-8.63） | 4.71（30.77） | 2.19（0.80） | 4.55（2.49） |
|  | SARS-CoV-2 antibody test positive* | 7 | 5.81（2.71-12.43） | 5.80（26.35） | 2.47（0.51） | 5.55（2.59） |
|  | KL-6 increased* | 6 | 51.42（19.3-137） | 51.41（197.73） | 5.11（1.00） | 34.61（12.99） |
|  | Body surface area increased* | 6 | 18.70（7.83-44.62） | 18.70（85.03） | 4.00（0.86） | 15.97（6.69） |
|  | HLA-B*27 positive* | 5 | 9.18（3.68-22.92） | 9.18（33.46） | 3.09（0.31） | 8.51（3.41） |
| Musculoskeletal and connective tissue disorders | Enthesopathy* | 86 | 12.70（10.15-15.89） | 12.69（824.27） | 3.51（3.00） | 11.40（9.11） |
|  | Finger deformity* | 82 | 4.14（3.32-5.16） | 4.14（187.57） | 2.01（1.61） | 4.02（3.22） |
|  | Spondylitis | 68 | 4.94（3.87-6.30） | 4.94（203.80） | 2.25（1.79） | 4.76（3.73） |
|  | Dactylitis* | 61 | 15.31（11.70-20.03） | 15.30（709.66） | 3.75（3.07） | 13.45（10.27） |
|  | Rheumatic disorder | 55 | 5.70（4.35-7.48） | 5.70（202.03） | 2.45（1.91） | 5.45（4.16） |
|  | Sacroiliitis | 47 | 6.17（4.60-8.29） | 6.17（192.19） | 2.56（1.95） | 5.88（4.38） |
|  | Tenosynovitis | 39 | 3.51（2.55-4.83） | 3.51（67.54） | 1.78（1.18） | 3.42（2.49） |
|  | Plantar fasciitis | 36 | 2.86（2.05-3.99） | 2.86（42.40） | 1.49（0.89） | 2.81（2.02） |
|  | Joint lock | 35 | 2.97（2.12-4.15） | 2.97（44.37） | 1.54（0.93） | 2.91（2.08） |
|  | Spondyloarthropathy | 29 | 12.80（8.7-18.83） | 12.80（280.51） | 3.52（2.48） | 11.49（7.81） |
|  | Seronegative arthritis | 25 | 7.16（4.77-10.74） | 7.16（123.89） | 2.76（1.82） | 6.76（4.51） |
|  | Axial spondyloarthritis | 17 | 13.45（8.11-22.30） | 13.45（173.21） | 3.59（2.08） | 12.01（7.24） |
|  | Oligoarthritis | 9 | 13.04（6.52-26.08） | 13.03（88.75） | 3.55（1.33） | 11.68（5.84） |
|  | Lordosis | 7 | 5.37（2.51-11.49） | 5.37（23.67） | 2.37（0.45） | 5.15（2.41） |
|  | Ligament calcification* | 4 | 18.70（6.44-54.26） | 18.70（56.69） | 4.00（0.13） | 15.97（5.50） |
|  | Diffuse idiopathic skeletal hyperostosis* | 4 | 11.12（3.96-31.19） | 11.12（33.23） | 3.34（0.01） | 10.13（3.61） |
| Neoplasms benign, malignant and unspecified (incl cysts and polyps) | Cutaneous T-cell lymphoma* | 25 | 3.41（2.29-5.09） | 3.41（41.30） | 1.74（0.97） | 3.34（2.24） |
|  | Acral lentiginous melanoma* | 4 | 22.85（7.73-67.52） | 22.85（68.38） | 4.24（0.16） | 18.88（6.39） |
| Nervous system disorders | Bell's palsy* | 10 | 6.05（3.20-11.45） | 6.05（39.8） | 2.53（0.93） | 5.77（3.05） |
|  | Morton's neuralgia | 7 | 4.56（2.14-9.71） | 4.56（18.60） | 2.14（0.32） | 4.40（2.07） |
| Pregnancy, puerperium and perinatal conditions | Normal newborn | 70 | 3.34（2.63-4.23） | 3.33（110.80） | 1.71（1.29） | 3.26（2.57） |
| Psychiatric disorders | Near death experience | 39 | 3.35（2.43-4.60） | 3.34（62.09） | 1.71（1.12） | 3.27（2.38） |
|  | Self-consciousness | 7 | 13.09（5.96-28.74） | 13.09（69.32） | 3.55（0.98） | 11.72（5.34） |
| Reproductive system and breast disorders | Genital ulceration | 14 | 5.76（3.36-9.87） | 5.76（52.13） | 2.46（1.19） | 5.51（3.21） |
| Respiratory, thoracic and mediastinal disorders | Oropharyngeal discomfort | 81 | 2.59（2.08-3.23） | 2.59（77.25） | 1.35（0.98） | 2.55（2.05） |
|  | Respiratory symptom | 34 | 2.93（2.09-4.13） | 2.93（42.12） | 1.53（0.91） | 2.88（2.05） |
|  | Tonsillar hypertrophy | 27 | 4.11（2.80-6.04） | 4.11（61.17） | 2.00（1.23） | 3.99（2.72） |
|  | Catarrh | 25 | 5.92（3.96-8.87） | 5.92（96.73） | 2.50（1.61） | 5.66（3.78） |
|  | Pharyngeal ulceration | 19 | 3.42（2.16-5.40） | 3.42（31.42） | 1.74（0.83） | 3.34（2.11） |
|  | Pharyngeal inflammation | 19 | 3.67（2.32-5.80） | 3.67（35.68） | 1.84（0.92） | 3.58（2.27） |
|  | Tonsillar ulcer | 13 | 19.10（10.57-34.52） | 19.10（188.02） | 4.02（1.99） | 16.26（9.00） |
|  | Nasal crusting | 13 | 4.40（2.52-7.66） | 4.40（32.72） | 2.09（0.86） | 4.26（2.44） |
|  | Allergic cough | 13 | 3.64（2.09-6.33） | 3.64（24.07） | 1.83（0.67） | 3.55（2.04） |
|  | Tonsillar inflammation | 11 | 9.05（4.89-16.76） | 9.05（72.38） | 3.07（1.35） | 8.40（4.53） |
|  | Oropharyngeal plaque | 10 | 5.75（3.04-10.86） | 5.74（37.11） | 2.46（0.89） | 5.49（2.91） |
|  | Xyphoid retraction | 4 | 37.39（11.91-117.44） | 37.39（103.89） | 4.79（0.18） | 27.69（8.82） |
| Skin and subcutaneous tissue disorders | Skin swelling | 96 | 2.57（2.10-3.14） | 2.57（89.50） | 1.34（1.00） | 2.53（2.06） |
|  | Nail psoriasis | 68 | 40.21（30.38-53.23） | 40.19（1868.23） | 4.87（3.96） | 29.17（22.04） |
|  | Onychomadesis* | 64 | 4.92（3.83-6.33） | 4.92（190.91） | 2.25（1.77） | 4.74（3.69） |
|  | Rebound psoriasis | 61 | 45.81（33.88-61.95） | 45.78（1848.96） | 5.00（3.97） | 31.99（23.65） |
|  | Skin hypertrophy | 57 | 5.68（4.35-7.41） | 5.67（208.01） | 2.44（1.92） | 5.43（4.16） |
|  | Dermatitis exfoliative generalised | 48 | 3.10（2.32-4.13） | 3.09（66.07） | 1.60（1.09） | 3.03（2.28） |
|  | Dandruff | 40 | 3.76（2.74-5.16） | 3.76（78.18） | 1.87（1.28） | 3.66（2.67） |
|  | Skin depigmentation | 39 | 7.32（5.29-10.13） | 7.32（198.61） | 2.79（2.07） | 6.90（4.98） |
|  | Erythrodermic psoriasis | 37 | 13.79（9.78-19.44） | 13.78（386.87） | 3.62（2.70） | 12.27（8.71） |
|  | Guttate psoriasis | 35 | 13.28（9.34-18.89） | 13.28（351.99） | 3.57（2.63） | 11.88（8.35） |
|  | Vitiligo* | 30 | 3.54（2.46-5.09） | 3.54（52.80） | 1.79（1.10） | 3.45（2.40） |
|  | Dermatitis psoriasiform | 29 | 4.07（2.81-5.90） | 4.07（64.56） | 1.98（1.25） | 3.95（2.73） |
|  | Skin weeping | 23 | 3.58（2.36-5.43） | 3.58（41.40） | 1.81（0.99） | 3.50（2.31） |
|  | Onycholysis* | 23 | 3.87（2.55-5.86） | 3.86（47.07） | 1.91（1.08） | 3.76（2.48） |
|  | Onychalgia | 19 | 4.44（2.81-7.03） | 4.44（48.55） | 2.10（1.13） | 4.30（2.72） |
|  | Nail pitting | 19 | 13.86（8.58-22.38） | 13.86（199.73） | 3.62（2.20） | 12.33（7.64） |
|  | Dyshidrotic eczema | 18 | 8.41（5.20-13.61） | 8.41（108.69） | 2.97（1.75） | 7.85（4.86） |
|  | Skin oedema | 16 | 4.55（2.75-7.50） | 4.54（42.37） | 2.14（1.05） | 4.39（2.66） |
|  | Palmoplantar pustulosis | 15 | 7.91（4.68-13.38） | 7.91（84.08） | 2.89（1.54） | 7.42（4.39） |
|  | Ingrown hair* | 13 | 4.56（2.62-7.95） | 4.56（34.62） | 2.14（0.90） | 4.41（2.53） |
|  | Stasis dermatitis | 12 | 5.12（2.87-9.14） | 5.12（37.90） | 2.30（0.95） | 4.92（2.76） |
|  | Granuloma annulare* | 12 | 4.39（2.46-7.83） | 4.39（30.14） | 2.09（0.80） | 4.25（2.39） |
|  | Nail dystrophy* | 11 | 5.92（3.23-10.88） | 5.92（42.55） | 2.5（1.01） | 5.65（3.08） |
|  | Nail bed disorder* | 11 | 5.55（3.02-10.17） | 5.54（38.88） | 2.41（0.95） | 5.31（2.90） |
|  | Intertrigo | 10 | 5.36（2.84-10.11） | 5.36（33.67） | 2.36（0.83） | 5.14（2.72） |
|  | Nail hypertrophy* | 7 | 12.20（5.57-26.71） | 12.20（64.34） | 3.46（0.95） | 11.01（5.03） |
|  | Nail bed inflammation* | 7 | 5.67（2.65-12.13） | 5.67（25.50） | 2.44（0.49） | 5.42（2.53） |
|  | Hand dermatitis | 7 | 6.10（2.85-13.08） | 6.10（28.18） | 2.54（0.55） | 5.81（2.71） |
|  | Eczema nummular | 6 | 4.78（2.11-10.84） | 4.78（17.15） | 2.21（0.18） | 4.61（2.04） |
|  | Acanthosis* | 6 | 7.43（3.25-17.02） | 7.43（31.15） | 2.81（0.48） | 7.00（3.06） |
|  | Leukoplakia | 5 | 7.45（3.01-18.47） | 7.45（26.04） | 2.81（0.21） | 7.02（2.83） |
| Vascular disorders | Aortic elongation* | 4 | 18.70（6.44-54.26） | 18.70（56.69） | 4.00（0.13） | 15.97（5.50） |

*Emerging findings of secukinumab associated AEs from FAERS database. ROR, reporting odds ratio; CI, confidence interval; PRR, proportional reporting ratio; χ^2^, chi-squared; IC, information component; EBGM, empirical Bayesian geometric mean.

**Supplementary Table 3.** **Signal strength of reports unrelated to secukinumab at the Preferred Term (PT) level in FAERS database.**

| SOC | Preferred Terms (PTs) | Secukinumab Cases  Reporting PT | ROR  (95% two-sided CI) | PRR (χ2) | IC (IC025) | EBGM (EBGM05) |
| --- | --- | --- | --- | --- | --- | --- |
| Injury, poisoning and procedural complications | Inappropriate schedule of product administration | 4616 | 4.38（4.25-4.52） | 4.21（10975.66） | 2.03（1.98） | 4.08（3.96） |
|  | Incorrect dose administered | 3239 | 4.23（4.08-4.38） | 4.11（7398.19） | 2.00（1.94） | 3.99（3.85） |
|  | Accidental exposure to product | 967 | 2.59（2.43-2.77） | 2.58（913.54） | 1.34（1.25） | 2.54（2.38） |
|  | Incorrect route of product administration | 714 | 3.63（3.37-3.91） | 3.61（1302.86） | 1.82（1.70） | 3.52（3.26） |
|  | Product prescribing error | 646 | 3.05（2.82-3.30） | 3.04（860.62） | 1.58（1.45） | 2.98（2.76） |
|  | Incorrect dose administered by device | 408 | 5.09（4.61-5.63） | 5.07（1273.10） | 2.29（2.12） | 4.88（4.42） |
|  | Wound | 373 | 3.59（3.24-3.98） | 3.58（670.40） | 1.80（1.64） | 3.49（3.15） |
|  | Scratch | 182 | 5.08（4.37-5.89） | 5.07（566.76） | 2.29（2.03） | 4.88（4.20） |
|  | Arthropod bite | 91 | 3.02（2.46-3.73） | 3.02（119.67） | 1.57（1.21） | 2.96（2.41） |
|  | Skin injury | 55 | 3.58（2.73-4.68） | 3.57（98.59） | 1.80（1.32） | 3.49（2.67） |
|  | Animal bite | 39 | 3.87（2.81-5.33） | 3.87（80.04） | 1.91（1.31） | 3.77（2.74） |
|  | Maternal exposure timing unspecified | 35 | 3.84（2.74-5.38） | 3.84（70.78） | 1.90（1.26） | 3.73（2.66） |
|  | Device dispensing error | 34 | 8.99（6.33-12.77） | 8.99（221.97） | 3.06（2.23） | 8.35（5.88） |
|  | Wrong schedule | 32 | 5.71（4.00-8.16） | 5.71（117.86） | 2.45（1.70） | 5.46（3.83） |
|  | Skin wound | 32 | 5.09（3.57-7.26） | 5.09（100.09） | 2.29（1.56） | 4.89（3.43） |
|  | Exposure via partner | 27 | 8.96（6.05-13.28） | 8.96（175.54） | 3.06（2.09） | 8.32（5.61） |
|  | Injection related reaction | 24 | 4.28（2.84-6.44） | 4.28（57.86） | 2.05（1.22） | 4.15（2.76） |
|  | Arthropod sting | 22 | 3.27（2.14-5.01） | 3.27（33.67） | 1.68（0.86） | 3.20（2.10） |
|  | Paternal exposure during pregnancy | 20 | 6.79（4.32-10.67） | 6.79（92.58） | 2.68（1.62） | 6.43（4.09） |
|  | Epicondylitis | 20 | 3.53（2.26-5.52） | 3.53（35.13） | 1.79（0.90） | 3.45（2.21） |
|  | Exposure to SARS-CoV-2 | 18 | 3.89（2.43-6.23） | 3.89（37.22） | 1.92（0.95） | 3.78（2.36） |
|  | Ear injury | 14 | 6.40（3.73-10.98） | 6.40（60.03） | 2.60（1.29） | 6.08（3.54） |
|  | Nail injury | 13 | 4.97（2.85-8.67） | 4.97（39.32） | 2.26（0.99） | 4.79（2.74） |
|  | Duplicate therapy error | 11 | 9.12（4.92-16.90） | 9.12（73.06） | 3.08（1.35） | 8.46（4.57） |
|  | Palate injury | 7 | 23.22（10.22-52.73） | 23.22（121.42） | 4.26（1.17） | 19.13（8.42） |
| Product issues | Device malfunction | 1379 | 5.55（5.26-5.86） | 5.48（4810.33） | 2.39（2.31） | 5.25（4.98） |
|  | Device issue | 994 | 2.28（2.14-2.43） | 2.27（690.94） | 1.16（1.07） | 2.24（2.10） |
|  | Needle issue | 374 | 3.22（2.90-3.56） | 3.21（551.39） | 1.65（1.49） | 3.14（2.83） |
|  | Device defective | 153 | 5.17（4.40-6.08） | 5.16（489.45） | 2.31（2.03） | 4.97（4.22） |
|  | Product availability issue | 146 | 2.78（2.35-3.27） | 2.77（161.25） | 1.45（1.18） | 2.73（2.31） |
|  | Product distribution issue | 108 | 7.41（6.10-9.01） | 7.40（558.09） | 2.80（2.42） | 6.97（5.74） |
|  | Product supply issue | 66 | 2.90（2.27-3.71） | 2.90（80.08） | 1.51（1.09） | 2.85（2.23） |
|  | Manufacturing product shipping issue | 15 | 4.63（2.76-7.77） | 4.63（40.88） | 2.16（1.02） | 4.48（2.67） |
|  | Manufacturing product storage issue | 7 | 13.58（6.17-29.87） | 13.58（72.06） | 3.60（1.00） | 12.11（5.51） |
| Social circumstances | Insurance issue | 106 | 3.17（2.61-3.85） | 3.17（152.75） | 1.63（1.31） | 3.10（2.56） |
| Surgical and medical procedures | Therapy interrupted | 275 | 2.52（2.24-2.84） | 2.52（245.91） | 1.31（1.12） | 2.48（2.20） |

ROR, reporting odds ratio; CI, confidence interval; PRR, proportional reporting ratio; χ^2^, chi-squared; IC, information component; EBGM, empirical Bayesian geometric mean.
